# Immunogenicity and Antibody Persistence of the Inactivated Quadrivalent Influenza Vaccine in Pediatric Patients Post-Chemotherapy or Allogeneic Hematopoietic Stem Cell Transplantation Versus Healthy Controls

**DOI:** 10.3390/vaccines12111224

**Published:** 2024-10-28

**Authors:** Mi Yeon Hur, Kyu Ri Kang, Ye Ji Kim, Yoon Kyung Cho, Jae Wook Lee, Nack-Gyun Chung, Bin Cho, Dae Chul Jeong, Jin Han Kang, Hyun Mi Kang

**Affiliations:** 1 Vaccine Bio Research Institute, College of Medicine, Catholic University of Korea, Seoul 06591, Republic of Korea; 2 Department of Pediatrics, College of Medicine, Catholic University of Korea, Seoul 06591, Republic of Korea

**Keywords:** influenza, vaccine, hematopoietic stem cell transplantation, chemotherapy, children

## Abstract

Pediatric patients who have undergone hematopoietic stem cell transplantation (HSCT) or chemotherapy are at increased risk for severe influenza complications, necessitating annual vaccination. This study evaluated the immunogenicity and antibody persistence of the 2021–2022 seasonal quadrivalent influenza vaccine in pediatric patients post-HSCT or chemotherapy, compared to healthy controls. A prospective cohort study included 80 pediatric participants divided into three groups: chemotherapy (*n* = 33), HSCT (*n* = 27), and healthy controls (*n* = 20). All participants were vaccinated with the 2021–2022 GC FLU Quadrivalent vaccine. Hemagglutination inhibition (HI) assays measured seroprotection rates (SPR), geometric mean titers (GMT), and seroconversion rates (SCR) for the four vaccine antigens (A/H1N1, A/H3N2, B/Victoria, B/Yamagata) at one, three, and six months post-vaccination. At one month post-vaccination, all groups met the 70% SPR threshold for A/H1N1 and A/H3N2, but not for B/Victoria. For B/Yamagata, the SPR was low in the chemotherapy and HSCT groups (18.18% and 33.33%, respectively), compared to 80.00% in controls (*p* < 0.0001 and *p* = 0.0015). While A/H1N1 and A/H3N2 GMTs were protective in all groups, only controls achieved protective levels for B/Yamagata. Over time, the control group maintained >70% SPR for A/H1N1 up to six months, but the chemotherapy and HSCT groups declined by three and six months, respectively. For A/H3N2, the SPR in controls dropped below 70% at three months, while it remained above 70% in the chemotherapy and HSCT groups until three months. None of the groups achieved protective GMTs for B strains at three or six months. Pediatric patients post-HSCT or chemotherapy demonstrated a comparable immune response to healthy controls for A/H1N1 and A/H3N2, but the rapid decline in A/H1N1 antibody levels suggests the need for ongoing monitoring and adjusted vaccination schedules. The poor response to B antigens, particularly B/Yamagata, underscores the need for improved vaccination strategies in these vulnerable populations.

## 1. Introduction

Pediatric patients who have undergone hematopoietic stem cell transplantation (HSCT) are at a significantly higher risk for severe complications and adverse outcomes compared to their healthy peers. As a result, these patients are prioritized for annual influenza vaccination, with current guidelines recommending the initiation of vaccination six months post-transplant [1,2].

The immunogenicity of the influenza vaccine in healthy children typically results in a robust and protective immune response, characterized by the production of neutralizing antibodies that target the four strains included in the inactivated quadrivalent influenza vaccine (A/H1N1, A/H3N2, B/Victoria, B/Yamagata) [3,4]. In contrast, children who have undergone hematopoietic stem cell transplantation (HSCT) often exhibit a more variable and sometimes diminished response to the vaccine [5,6]. This difference is largely attributed to the prolonged recovery of the immune system post-transplant, particularly the delayed reconstitution of B-cells, which are critical for effective antibody production. Furthermore, the use of immunosuppressive therapies to prevent graft-versus-host disease (GvHD) in HSCT patients can further impair vaccine efficacy [7], making this population less likely to achieve the same level of seroprotection as their healthy counterparts. Despite these challenges, studies have shown that, with time, pediatric HSCT recipients can mount a sufficient immune response to the influenza vaccine, although the timing and duration of antibody persistence may differ from those seen in healthy children [8,9].

While previous studies in adult HSCT recipients have demonstrated that influenza vaccination offers clinical benefits, including lower infection rates and reduced hospitalizations [8,10], data on pediatric HSCT recipients remain sparse. Furthermore, significant gaps persist in our understanding of the effectiveness and longevity of antibody responses following influenza vaccination in children, particularly those who have undergone HSCT or chemotherapy. To address these gaps, it is essential to gather foundational data on the immunogenicity and antibody persistence associated with the four influenza vaccine antigens (A/H1N1, A/H3N2, B/Victoria, B/Yamagata) in pediatric patients who have undergone HSCT or chemotherapy. Therefore, the primary objective of this study was to evaluate the immunogenicity of the first influenza vaccine administered after hematopoietic stem cell transplantation and compare it with the immune response observed in children who received the vaccine during or immediately following the final cycle of chemotherapy, as well as with that of healthy controls. The secondary objective was to assess the persistence of vaccine-induced antibodies at three and six months post-vaccination across these groups.

## 2. Methods

### 2.1. Study Participants

This prospective cohort study involved children below 19 years of age who were vaccinated with the seasonal inactivated influenza vaccine (GC FLU Quadrivalent, GC Biopharma, Yongin, Republic of Korea) during the 2021–2022 influenza season. Participants were divided into three groups: the chemotherapy group, the HSCT group, and a healthy control group. The chemotherapy group consisted of patients who received their first influenza vaccine after the diagnosis of their malignancy and initiation of treatment. This included patients vaccinated during maintenance chemotherapy cycles or those who received their first influenza vaccine immediately after completing therapy. The HSCT group included those who had undergone hematopoietic stem cell transplantation at Seoul St. Mary’s Hospital and received their first influenza vaccine post-transplant. The control group comprised children with no history of immunocompromising diseases or immunosuppressant use. The inclusion criteria for all groups were as follows: (1) documented verification of the vaccine and vaccination date, and (2) no contraindications for influenza vaccination. This study received approval from the Institutional Review Board (IRB) of Seoul St. Mary’s Hospital (KC21TASI0182).

### 2.2. Study Vaccines

Patients in this study were immunized with the 2021–2022 seasonal inactivated influenza vaccine (GC FLU Quadrivalent, GC Biopharma Corp.). The vaccine contained the following strains from the 2021–2022 northern hemisphere: A/Victoria/2570/2019 (A/H1N1), A/Cambodia/e0826360/2020 (A/H3N2), B/Washington/02/2019 (B/Victoria lineage), and B/Phuket/3073/2013 (B/Yamagata lineage). These strains were cultivated in eggs, inactivated, and split, with one single-dose vial of 0.5 mL containing 15 μg of each of the four purified hemagglutinin antigens (60 μg total). Immunization was administered according to the 2021–2022 Advisory Committee on Immunization Practices and Korea Disease Control and Prevention Agency recommendation guidelines [11,12].

### 2.3. Study Design

Blood samples were collected from each of the patients in the chemotherapy, HSCT, and control groups before vaccination and one month after vaccination to assess SCR, SPR, GMT, and GMR. Additional blood samples were collected at 3 and 6 months post-vaccination to assess antibody persistence by measuring changes in SPR, GMT, and GMR over time.

### 2.4. Hemagglutination Inhibition (HI) Assay

The hemagglutination inhibition (HI) assay was conducted in accordance with the WHO guidelines [13]. Serum samples were treated with receptor destroying enzyme (RDE) from Vibrio cholerae Ogawa type 558 (Cosmos Biomedical Ltd., Derbyshire, UK) at a 1:3 ratio, incubated overnight at 37 °C, then boiled at 56 °C for 30 min. After adding saline to adjust the volume, turkey red blood cells were added to remove non-specific reactions, and the supernatant serum was used for the assay. Standard antigens corresponding to the 2021–2022 northern hemisphere influenza vaccine virus strains were diluted to 4 HA units/25 μL, and the treated serum samples were reacted in a V-bottom 96-well plate (Corning Costar Cat No. 3894). After adding 0.5% turkey red blood cells (Innovative Research, Novi, MI, USA) and incubating for 30 min, the HI titer was determined by reading the highest dilution at which red blood cells settled completely in the negative control well and did not flow when the plate was tilted. The reciprocal of the highest dilution of serum where inhibition of hemagglutination appeared by tear drop of RBCs was recorded as the HI titer.

An HI titer of 1:40 or greater was considered seropositive. Using the results of the HI assay, the geometric mean titer (GMT), geometric mean ratio (GMR), seropositive rate (SPR), and seroconversion rate (SCR) were calculated for each group. The GMR was determined as the ratio of post-vaccination to pre-vaccination GMT. Seroconversion was defined as either a pre-vaccination HI titer of ≤1:10 increasing to a post-vaccination HI titer of ≥1:40, or as a fourfold or greater increase in the HI titer from pre-vaccination to post-vaccination. The SCR was calculated as the percentage of patients that met the criteria for seroconversion at one month post-vaccination. The SPR was defined as the percentage of individuals with a post-vaccination titer of ≥1:40. The criteria for these parameters were based on guidelines from the Center for Biologics Evaluation and Research (CBER) and the Committee for Medicinal Products for Human Use (CHMP) (10, 11), in which the SCR licensure criteria for the HI assay in subjects under 65 years old are ≥40% (lower bound of two-sided 95% CI) for CBER and >40% for CHMP. The SPR criteria are ≥70% (lower bound of two-sided 95% CI) for CBER and >70% for CHMP (Table S1).

### 2.5. Statistical Analysis

Categorical variables were compared using the chi-square test. The Kruskal–Wallis one-way ANOVA was employed to identify statistically significant differences in continuous variables across the three groups. The GMT of the HI titer and the two-sided 95% confidence intervals (CI) were calculated using GraphPad Prism™ software v9.4.1 (San Diego, CA, USA). The GMR and its two-sided 95% CI were also calculated using the paired t-test formula in GraphPad Prism™ software v9.4.1 (San Diego, CA, USA). Additionally, the percentage, as well as the upper and lower limits of SPR and SCR, were determined using GraphPad Prism™. All statistical tests were two-sided, with significance levels set at * *p* < 0.05, ** *p* < 0.01, *** *p* < 0.001, and **** *p* < 0.0001.

## 3. Results

### 3.1. Patient Characteristics

A total of 80 participants were included in this study, with the following group distributions: 41.3% (*n* = 33) in the chemotherapy group, 33.8% (*n* = 27) in the HSCT group, and 25.0% (*n* = 20) in the control group. The mean age at influenza vaccination was as follows: 10.1 years (standard deviation [SD] ± 3.8) in the chemotherapy group, 10.0 years (SD ± 5.3) in the HSCT group, and 9.5 years (SD ± 4.1) in the control group (*p* = 0.734) (Table 1).

### 3.2. Immunogenicity Analyses

**Figure 1:**
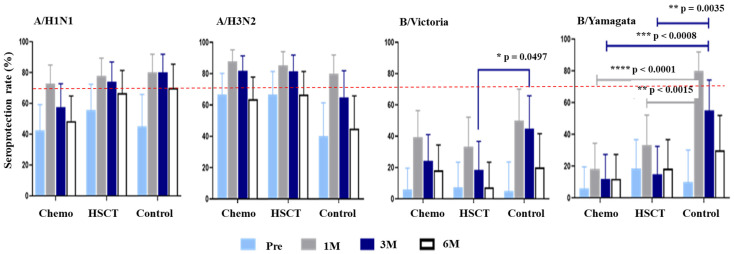
Seroprotection rates at pre-vaccination and post-vaccination 1, 3, and 6 months in the chemotherapy, hematopoietic stem cell transplantation, and control groups. Abbreviations: Chemo, chemotherapy; HSCT, hematopoietic stem cell transplantation; pre, pre-vaccination; M, months. Statistical significance was defined as * *p* < 0.05; ** *p* < 0.01; *** *p* < 0.001; **** *p* < 0.0001.

The proportion of patients with pre-vaccination seropositive HI titers was ≥40% for the A/H1N1 and A/H3N2 antigens across all three groups. However, for the B/Victoria and B/Yamagata strains, the pre-vaccination SPR was below 20% (Figure 1, Table S2). In a subanalysis exploring the unexpectedly high pre-vaccination titers (≥40%) for A antigens in patients who underwent HSCT—despite being considered immunologically naïve due to this being their first vaccination post-transplant—we examined the impact of different conditioning regimens. Specifically, we compared patients who received myeloablative conditioning with those who received reduced-intensity conditioning prior to HSCT. For the A/H1N1 antigen, the pre-vaccination SPR was significantly higher in the reduced-intensity group (100%, *n* = 5/5) compared to the myeloablative group (45.5%, *n* = 10/23; *p* = 0.027) (Table 2).

Despite this, the GMTs for both the A and B antigens were below 40 in all three groups, except for A/H3N2, where the GMT in the chemotherapy group was 40.85 (95% confidence interval [CI], 29.1–57.35) and in the HSCT group was 55.85 (95% CI, 35.53–87.79); however, this difference was statistically insignificant when compared to the control group (*p* = 0.789 and *p* = 0.189, respectively) (Table S3).

**Figure 2:**
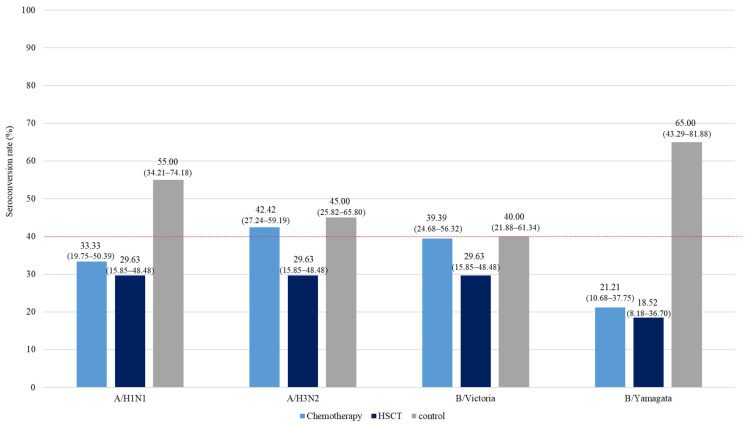
Seroconversion rates at pre-vaccination and post-vaccination 1, 3, and 6 months in the chemotherapy, hematopoietic stem cell transplantation, and control groups. Conversion rates for each group are shown above the bars. The lower and upper bound 95% confidence intervals are written within the parentheses. Abbreviations: HSCT, hematopoietic stem cell transplantation.

At one month post-vaccination, the SCR in both the chemotherapy and HSCT groups was below the 40% threshold set by CBER and CHMP, with one exception: the A/H3N2 strain in the chemotherapy group, where the SCR exceeded 40%, reaching 42.42% (95% CI, 27.24–59.19). The HSCT group did not meet the 40% threshold for any of the four strains. In contrast, the control group met the CHMP SCR criteria of >40% for A/H1N1, A/H3N2, and B/Yamagata (Figure 2).

The SPR at one month post-vaccination in all three groups met the CHMP SPR criteria of greater than 70% for the A/H1N1 and A/H3N2 strains. For the B/Victoria strain, all three groups had an SPR below 70%. For the B/Yamagata strain, the SPR was particularly low in both the chemotherapy and HSCT groups, at 18.18% (95% CI, 8.61–34.39) and 33.33% (95% CI, 18.64–52.18), respectively. In contrast, the control group had a significantly higher SPR for the B/Yamagata strain, reaching 80.00% (95% CI, 58.40–91.93), and this difference was statistically significant (*p* < 0.0001 and *p* = 0.0015, respectively) (Figure 1, Table S2).

**Figure 3:**
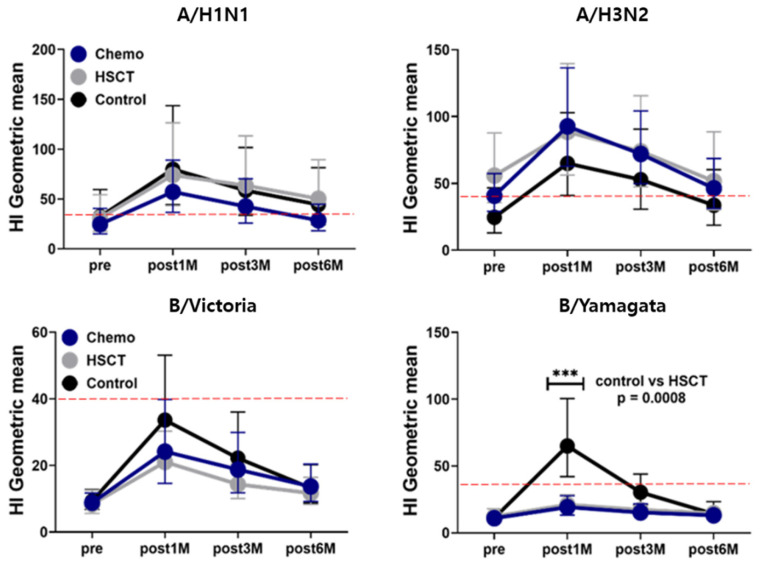
Hemagglutinin inhibition geometric mean titer rates at pre-vaccination and post-vaccination 1, 3, and 6 months in the chemotherapy, hematopoietic stem cell transplantation, and control groups. For B/Yamagata, the gray line representing the HSCT group closely overlaps with the blue line for the chemotherapy group due to their very similar titer responses. Abbreviations: Chemo, chemotherapy; HSCT, hematopoietic stem cell transplantation; pre, pre-vaccination; M, months.

At one month post-vaccination, the GMTs for A/H1N1 and A/H3N2 exceeded 1:40 in all three groups. However, for the B strains, only the control group reached a GMT greater than 1:40 for B/Yamagata, with a GMT of 64.98 (95% CI, 42.05–100.40), which was significantly higher compared to the HSCT group’s GMT of 21.05 (95% CI, 15.54–28.52) (*p* = 0.0008). For B/Victoria, the GMTs in all groups remained below the seropositive threshold of 40 (Figure 3, Table S3). When comparing the geometric mean ratios (GMRs) at one month post-vaccination, the control group met the CHMP criteria of greater than 2.5 for A/H3N2, B/Victoria, and B/Yamagata, with GMRs of 2.64 (95% CI, 1.66–4.20), 3.61 (95% CI, 2.06–6.31), and 5.86 (95% CI, 3.44–9.96), respectively. However, for A/H1N1, the GMR was slightly below the criteria, at 2.46 (95% CI, 1.59–3.81). In the chemotherapy and HSCT groups, only the GMR for B/Victoria exceeded 2.5, with values of 2.74 (95% CI, 1.66–4.52) in the chemotherapy group and 2.52 (95% CI, 1.63–3.90) in the HSCT group (Table 3).

### 3.3. Duration of Antibodies

#### 3.3.1. A/H1N1 Antigen Response

In the control group, the SPR for the A/H1N1 antigen was sustained at 80.00% (95% CI: 58.4–91.93) at 3 months post-vaccination, with a slight decrease to 70.00% (95% CI: 48.10–85.45) at 6 months. In the HSCT group, the SPR remained above the 70% threshold, starting at 77.78% (95% CI: 59.24–89.39) at 1 month post-vaccination and slightly declining to 74.07% (95% CI: 55.32–86.83) at 3 months, before further decreasing to 66.67% (95% CI: 47.82–81.36) at 6 months. In contrast, the chemotherapy group experienced a significant decline in SPR, falling below the 70% threshold to 57.58% (95% CI: 40.81–72.76) by 3 months post-vaccination, and further declining to 48.48% (95% CI: 32.50–64.78) by 6 months (Table S2).

Despite these declines, the HI assay GMTs for all groups remained above 40 until 6 months post-vaccination, with the exception of the chemotherapy group, where the GMT decreased to an unprotective level of 28.58 (95% CI: 18.26–44.73) (Table S3).

#### 3.3.2. A/H3N2 Antigen Response

For the A/H3N2 antigen, the control group’s SPR dropped below the 70% threshold by 3 months post-vaccination, declining from 80.00% (95% CI: 54.40–91.93) at 1 month post-vaccination to 65.00% (95% CI: 43.29–81.88) at 3 months (Figure 1, Table S2). The HI GMTs, above the protective level at 3 months, decreased from 52.78 (95% CI: 30.73–90.64) to below the protective level at 33.64 (95% CI: 18.74–60.37) at 6 months (Figure 2, Table S3).

In both the chemotherapy and HSCT groups, the SPRs remained above the 70% threshold at 3 months post-vaccination, with values of 87.88% (95% CI: 72.67–95.18) and 85.19% (95% CI: 67.52–94.08), respectively. However, by 6 months, these rates had fallen below the 70% threshold, to 63.64% (95% CI: 46.62–77.81) in the chemotherapy group and 66.67% (95% CI: 47.82–81.36) in the HSCT group (Figure 1, Table S2). Notably, the GMTs in both groups remained above the protective level of 1:40 at 6 months, at 46.34 (95% CI: 31.3–68.59) for the chemotherapy group and 51.71 (95% CI: 30.18–88.6) for the HSCT group (Figure 2, Table S3).

#### 3.3.3. B Antigen Response

For the B/Yamagata antigen, the control group exhibited an SPR of 55.00% (95% CI: 47.82–81.36) at 3 months post-vaccination, which was significantly higher than that of the chemotherapy group (SPR of 12.12% [95% CI: 4.82–27.33], *p* = 0.0008) and the HSCT group (SPR of 14.81% [95% CI: 5.92–32.48], *p* = 0.0035) (Figure 1, Table S2). However, the GMT in the control group was 30.31 (95% CI: 20.93–43.91), which is below the protective cutoff level of 1:40. By 3 and 6 months post-vaccination, none of the groups, including the control, had achieved protective GMT levels for both the B/Victoria and B/Yamagata antigens.

## 4. Discussion

This study aimed to evaluate the immunogenicity and duration of antibody responses to the 2021–2022 seasonal influenza vaccine in pediatric patients who had undergone hematopoietic stem cell transplantation (HSCT) or chemotherapy, compared to healthy age-matched controls. The study included 80 participants divided into three groups: chemotherapy (41.3%), HSCT (33.8%), and control (25.0%). The analysis revealed that pre-vaccination seropositive rates were above 40% for the A/H1N1 and A/H3N2 antigens, but below 20% for the B/Victoria and B/Yamagata strains across all groups. One month post-vaccination, all groups met the CHMP SPR and GMT criteria for the A/H1N1 and A/H3N2 antigens, whereas the response to the B strains was significantly lower. Over time, the durability of the antibody response varied between groups. For the A/H1N1 antigen, the control group maintained an SPR above 70% for six months, while the chemotherapy group’s SPR dropped below 70% by three months, and the HSCT group’s SPR fell just below 70% by six months. However, only the chemotherapy group’s GMT fell below 1:40 by six months. For A/H3N2, the control group’s SPR dropped below 70% by three months, with the chemotherapy and HSCT groups following by six months, though their GMTs generally remained protective. The response to the B antigen was weak across all groups, with none maintaining protective GMT levels for B/Yamagata or B/Victoria at three months.

The observation that pre-vaccination seropositive rates exceeded 40% for the A/H1N1 and A/H3N2 antigens but remained below 20% for the B/Victoria and B/Yamagata strains can be explained by several factors. The higher pre-vaccination seropositive rates for A/H1N1 and A/H3N2 likely reflect greater population exposure due to previous infections and vaccinations. Influenza A viruses, particularly H1N1 and H3N2, have been more prevalent in recent influenza seasons, contributing to higher baseline immunity [14]. Conversely, influenza B viruses, especially the B/Yamagata lineage, have circulated less frequently in recent years, resulting in lower baseline immunity, as reflected in the lower pre-vaccination seropositive rates. However, it is important to interpret the effects of higher baseline titers with caution. Some studies have shown that repeated vaccination can negatively impact the hemagglutinin antibody response, particularly for H3N2 [15]. Since the median age of the patients was 9–10 years across all three groups, the majority had prior exposure, whether through natural infection or previous vaccination. Although HSCT patients were considered naive because this was their first vaccination post-HSCT, their pre-vaccination seropositive rates were similar to those of the chemotherapy and control groups.

A subanalysis of our data revealed that the type of conditioning regimen—myeloablative versus reduced-intensity—used in HSCT patients affected their pre-vaccination seroprotection rates (SPR). This indicates that the intensity and effects of these regimens on a patient’s bone marrow and immune system may influence vaccine response after HSCT. Additionally, a study on influenza vaccination in bone marrow transplant patients found that more aggressive conditioning regimens were associated with a higher risk of influenza [9], highlighting the need for vigilant prophylaxis in this group. Therefore, further research is needed to understand how conditioning regimens might interfere with or enhance the immune response to influenza vaccination in HSCT patients.

At one month post-vaccination, all groups successfully met the CHMP criteria for SPR and GMT for the A/H1N1 and A/H3N2 antigens. This suggests that the vaccine was generally effective in eliciting an adequate immune response to these influenza A strains, regardless of the participants’ underlying health conditions. However, the response to the influenza B strains, particularly B/Yamagata, was markedly weaker across the groups. While the control group met the SPR threshold and achieved protective GMT levels for the B/Yamagata strain at one month post-vaccination, the chemotherapy and HSCT groups did not. This disparity indicates a potentially reduced ability to mount a sufficient immune response to influenza B strains in immunocompromised patients, particularly those undergoing chemotherapy or HSCT. The lower response in the chemotherapy and HSCT groups could be attributed to their compromised immune systems, which may not have responded as robustly to the vaccine, especially against strains they had less prior exposure to, such as B/Yamagata [16,17]. This difference underscores the need for additional protective measures for immunocompromised populations, who may remain vulnerable to certain strains despite vaccination.

Over time, the durability of the antibody response varied between groups. For the A/H1N1 antigen, the control group maintained an SPR above 70% for six months, while the chemotherapy group’s SPR dropped below 70% by three months, and the HSCT group’s SPR fell just below 70% by six months. Only the chemotherapy group’s GMT fell below 1:40 by six months. Studies have shown that the efficacy of the influenza vaccine in patients undergoing chemotherapy is lower compared to the general population. While both patient groups exhibit reduced seroconversion rates compared to healthy individuals, HSCT patients typically have lower rates than those receiving chemotherapy alone. For example, studies found SCRs of around 40–60% in chemotherapy patients versus 20–40% in HSCT recipients [6,18,19,20,21]. This study found that the duration of protection against A/H1N1 in patients undergoing chemotherapy or post-HSCT may be reduced compared to healthy children by 3 to 6 months. Therefore, the timing of vaccination is important; administering the vaccine in September or October could result in insufficient antibody levels by the peak of the influenza season in December, potentially leaving these patients unprotected when they are most vulnerable. This highlights the need for enhanced monitoring and possibly an adjusted vaccination schedule for vulnerable populations, such as children undergoing chemotherapy. On the other hand, the duration of protection against A/H3N2 remained above the threshold until 3 months post-vaccination, unlike the control group, in which the SPR decreased to below 70%.

The poor immunogenicity observed for B strains, particularly B/Yamagata, across all groups suggests that the current vaccine formulation may be less effective in eliciting a robust and sustained immune response in pediatric patients undergoing chemotherapy or HSCT. Given that these patients are at increased risk for influenza-related complications, this finding raises concerns about the adequacy of current vaccination strategies for this high-risk population. These data suggest a potential need for vaccine reformulation, increased dosage, or alternative vaccination strategies, such as booster doses, to enhance protection against B strains in these vulnerable groups.

Research on the duration of influenza vaccine protection in patients undergoing chemotherapy or HSCT is limited, which is concerning given their compromised immune systems. Our study found that, while the vaccine’s protective effect against Influenza A was generally maintained, H1N1 immunity waned more rapidly in chemotherapy patients, likely due to the cytotoxic effects that impair antibody production and durability [22]. Notably, HSCT patients sustained protective titers against Influenza A for up to six months, highlighting the need for further research with larger patient cohorts to better understand these dynamics.

This study’s limitations, including the relatively small sample size and the lack of an influenza outbreak during the study period, must be acknowledged. These factors may limit the generalizability of the findings and the ability to correlate immunogenicity with actual vaccine effectiveness. Future studies with larger sample sizes and real-world effectiveness data are needed to confirm these findings and refine vaccination strategies for these vulnerable populations.

In conclusion, this study demonstrates that, while the immunogenicity and duration of immunity against A/H1N1 and A/H3N2 antigens in pediatric patients undergoing chemotherapy or HSCT are generally comparable to those in healthy children, the decline in antibody levels, particularly for the A/H1N1 strain, suggests a need for ongoing monitoring and potential modifications to vaccination schedules. Antigenic content, pre-vaccination titers, and adjuvant type are critical factors influencing influenza vaccine potency, immunogenicity, and duration of immunity. In our study, we used standard influenza vaccines without focusing on these specific factors, though we acknowledge their importance, especially for immunocompromised patients like those undergoing HSCT. The poor response to B antigens, especially B/Yamagata, indicates a need for improvements in vaccine formulation or administration strategies to better protect this vulnerable population. These findings highlight the critical need to optimize vaccination timing and develop more immunogenic influenza vaccines, including the use of adjuvants, for immunocompromised patients. Additionally, clinical trial studies are necessary to evaluate the importance of increased doses or a two-dose regimen administered at a 4-week interval using the standard dose for this population. Ongoing research is essential to ensure that influenza prevention measures maximize protection, particularly in vulnerable populations like those undergoing chemotherapy or HSCT.

## Figures and Tables

**Table 1:** Demographics of the patients included in this study.

	Chemotherapy *n* = 33	HSCT *n* = 27	Control *n* = 20	*p*
Male sex	14 (42.4)	12 (44.4)	12 (60.0)	0.760
Age	10.1 (±3.8)	10.0 (±5.3)	9.5 (±4.1)	0.734
No. of patients that received 2 doses	0	4 (14.8)	0	-
Underlying disease				<0.001
Acute lymphoid leukemia	27 (81.8)	9 (33.3)	-	
Acute myeloid leukemia	4 (12.1)	9 (33.3)	-	
JMML	-	3 (11.1)	-	
SAA	-	3 (11.1)	-	
Others	2 (6.1)	3 (11.1)	-	
None	-	-	20 (100)	

**Table 2:** Seroprotection rate of pre-vaccination HI titers for the four antigens, depending on the type of conditioning chemotherapy regimen in the HSCT group.

	No. of Cases (%)		
	Myeloablative (*n* = 22)	Reduced Intensity (*n* = 5)	*p*
A/H1N1	10 (45.5)	5 (100)	0.027
A/H3N2	13 (59.1)	5 (100)	0.080
B/Victoria	2 (9.1)	0 (0)	0.484
B/Yamagata	5 (22.7)	0 (0)	0.238

**Table 3:** Geometric mean ratio at one-month post-vaccination for each of the four antigens by group.

	Chemotherapy	HSCT	Control
A/H1N1	2.32 (1.45–3.70)	2.39 (1.37–4.18)	2.46 (1.59–3.81)
A/H3N2	2.27 (1.51–3.40)	1.59 (0.98–2.57)	**2.64 (1.66–4.20)**
B/Victoria	**2.74 (1.66–4.52)**	**2.52 (1.63–3.90)**	**3.61 (2.06–6.31)**
B/Yamagata	1.76 (1.28–2.43)	1.71 (1.24–2.37)	**5.86 (3.44–9.96)**

Geometric mean ratios exceeding the CHMP licensure criteria are shown in bold.

## Data Availability

Partial data (removing any identifiers) are available upon request from the corresponding author.

## Supplementary Materials

The following supporting information can be downloaded at: https://www.mdpi.com/article/10.3390/vaccines12111224/s1, Table S1: Center for Biologics Evaluation and Research (US), Committee for Medicinal Products for Human Use (European) licensure criteria for hemagglutinin inhibition assay in under 65 years old subjects; Table S2: Seroprotection rate and seroconversion rate (via HI assay) for each group at post-vaccination 1, 3, and 6 months; Table S3: Geometric mean titers of hemagglutinin inhibition assay results against 2021–2022 influenza strain for chemotherapy, hematopoietic stem cell transplantation, and control groups.
