# Supplementary material for: Immunogenicity and Antibody Persistence of the Inactivated Quadrivalent Influenza Vaccine in Pediatric Patients Post-Chemotherapy or Allogeneic Hematopoietic Stem Cell Transplantation Versus Healthy Controls
Source: Vaccines (Basel). 2024 Oct 28;12(11):1224. doi: 10.3390/vaccines12111224 (PMC11598254; doi:10.3390/vaccines12111224)
Supplement: Supplementary file 1 [file vaccines-12-01224-s001.zip › vaccines-3219448-supplementary.pdf]

Table S1. Center for Biologics Evaluation and Research (US), Committee for Medicinal Products for Human Use (European) licensure criteria for hemagglutinin inhibition assay in under 65 years old subjects

| | **CBER*** | **CHMP**** |
|---|---|---|
| SCR | ≥ 40%<br>(lower bound of two sided 95% CI) | > 40% |
| SPR | ≥ 70%<br>(lower bound of two sided 95% CI) | > 70% |
| GMR | | > 2.5 |
| SCR, seroconversion rate; SPR, seroprotection rate; GMR, geometric mean ratio | | |

Abbreviations: CBER, Center for Biologics Evaluation and Research; CHMP, Committee for Medicinal Products for Human Use

Table S2. Seroprotection rate and seroconversion rate (via HI assay) for each group at post-vaccination 1, 3, and 6 months

| Vaccine strain | Immune response | Months post vaccination | | | |
|---|---|---|---|---|---|
| A/H1N1 | | pre | Post1M | Post3M | Post6M |
| Chemotherapy | SPR% (95% CI) | 42.42 (27.24-59.19) | 72.73 (55.78-84.93) | 57.58 (40.81-72.76) | 48.48 (32.50-64.78) |
| | P value vs control | 0.8545 | 0.5508 | 0.0947 | 0.1255 |
| HSCT | SPR% (95% CI) | 55.56 (37.31-72.41) | 77.78 (59.24-89.39) | 74.07 (55.32-86.83) | 66.67 (47.82-81.36) |
| | P value vs control | 0.4741 | 0.8540 | 0.6352 | 0.8085 |
| Control | SPR% (95% CI) | 45.00 (25.82-65.79) | 80.00 (58.40-91.93) | 80.00 (58.4-91.93) | 70.00 (48.10-85.45) |
| Chemotherapy | SCR% (95% CI) | | 33.33 (19.75-50.39) | | |
| HSCT | | | 29.63 (15.85-48.48) | | |
| Control | | | 55.00 (34.21-74.18) | | |
| A/H3N2 | | pre | Post1M | Post3M | Post6M |
| Chemotherapy | SPR% (95% CI) | 66.67 (49.61-80.25) | 87.88 (72.67-95.18) | 81.82 (65.61-91.39) | 63.64 (46.62-77.81) |
| | P value vs control | 0.0576 | 0.4374 | 0.1678 | 0.1845 |
| HSCT | SPR% (95% CI) | 66.67 (47.82-81.36) | 85.19 (67.52-94.08) | 81.48 (63.30-91.82) | 66.67 (47.82-81.36) |
| | P value vs control | 0.0690 | 0.6400 | 0.2001 | 0.1374 |
| Control | | 40.00 (21.88-61.34) | 80.00 (58.40-91.93) | 65.00 (43.29-81.88) | 45.00 (25.82-65.8) |
| Chemotherapy | SCR% (95% CI) | | 42.42 (27.24-59.19) | | |
| HSCT | | | 29.63 (15.85-48.48) | | |
| Control | | | 45.00 (25.82-65.80) | | |
| B/Victoria | | pre | Post1M | Post3M | Post6M |
| Chemotherapy | SPR% (95% CI) | 6.06 (1.08-19.61) | 39.39 (24.68-56.32) | 24.24 (12.83-41.02) | 18.18 (8.61-34.39) |
| | P value vs control | 0.8713 | 0.4502 | 0.1166 | 0.8697 |
| HSCT | SPR% (95% CI) | 7.41 (1.32-23.37) | 33.33 (18.64-52.18) | 18.52 (8.18-36.70) | 7.41 (1.32-23.37) |
| | P value vs control | 0.7385 | 0.2497 | **0.0497** | 0.2009 |
| Control | SPR% (95% CI) | 5.00 (0.26-23.61) | 50.00 (29.93-70.07) | 45.00 (25.82-65.79) | 20.00 (8.07-41.60) |
| Chemotherapy | SCR% (95% CI) | | 39.39 (24.68-56.32) | | |
| HSCT | | | 29.63 (15.85-48.48) | | |
| Control | | | 40.00 (21.88-61.34) | | |
| B Yamagata | | pre | Post1M | Post3M | Post6M |
| Chemotherapy | SPR% (95% CI) | 6.06 (1.08-19.61) | 18.18 (8.61-34.39) | 12.12 (4.82-27.33) | 12.12 (4.82-27.33) |
| | P value vs control | 0.5987 | **<0.0001** | **0.0008** | 0.1068 |
| HSCT | SPR% (95% CI) | 18.52 (8.18-36.70) | 33.33 (18.64-52.18) | 14.81 (5.92-32.48) | 18.52 (8.18-36.70) |

| | | | | | |
|---|---|---|---|---|---|
| | P value vs control | 0.4174 | **0.0015** | **0.0035** | 0.3580 |
| Control | SPR% (95% CI) | 10 (1.78-30.1) | 80.00 (58.40-91.93) | 55.00 (34.21-74.18) | 30.00 (14.55-51.90) |
| Chemotherapy | SCR% (95% CI) | | 21.21 (10.68-37.75) | | |
| HSCT | | | 18.52 (8.18-36.70) | | |
| Control | | | 65.00 (43.29-81.88) | | |
| Chemo, Chemotherapy; CI, confidence interval; HSCT, Hematopoietic stem cell transplant; M, month; SPR, seroprotection rate; SCR, seroconversion rate | | | | | |

Abbreviations: SCR, seroconversion rate; SPR, seroprotection rate;

Chi square test was used to compare SPR of each test group, Chemo (n=33) and HSCT (n=27) with Control group (n=20). Statistical significance was defined as *p < 0.05; **p < 0.01; ***p < 0.001; ****p < 0.0001.

Table S3. Geometric mean titers of hemagglutinin inhibition assay results against 2021-2022 influenza strain for chemotherapy, hematopoietic stem cell transplantation, and control groups

| Vaccine strain | | Months post vaccination | | | |
|---|---|---|---|---|---|
| A/H1N1 | | pre | Post1M | Post3M | Post6M |
| Chemotherapy | GMT (95% CI) | 24.67 (15.01-40.57) | 57.17 (36.68-89.08) | 42.60 (25.82-70.28) | 28.58 (18.26-44.73) |
| | P value vs control | 0.6215 | 0.6262 | 0.8903 | 0.4449 |
| HSCT | GMT (95% CI) | 30.94 (17.59-54.43) | 74.07 (43.42-126.4) | 63.50 (35.58-113.3) | 50.40 (28.38-89.49) |
| | P value vs control | 0.6876 | 0.9970 | 0.7969 | 0.5424 |
| Control | GMT (95% CI) | 32.49 (17.73-59.53) | 80.00 (44.52-143.7) | 58.56 (33.73-101.7) | 44.38 (24.18-81.45) |
| A/H3N2 | | pre | Post1M | Post3M | Post6M |
| Chemotherapy | GMT (95% CI) | 40.85 (29.1-57.35) | 92.67 (62.91-136.5) | 72.02 (49.79-104.2) | 46.34 (31.3-68.59) |
| | P value vs control | 0.7894 | 0.3860 | 0.5441 | 0.8097 |
| HSCT | GMT (95% CI) | 55.85 (35.53-87.79) | 88.65 (56.28-139.6) | 74.07 (47.44-115.7) | 51.71 (30.18-88.6) |
| | P value vs control | 0.1890 | 0.3630 | 0.6086 | 0.4971 |
| Control | GMT (95% CI) | 24.62 (12.97-46.75) | 64.98 (41.02-102.9) | 52.78 (30.73-90.64) | 33.64 (18.74-60.37) |
| B Victoria | | Pre | Post1M | Post3M | Post6M |
| Chemotherapy | GMT (95% CI) | 8.82 (6.63-11.72) | 24.16 (14.65-39.84) | 18.78 (11.79-29.91) | 13.70 (9.20-20.42) |
| | P value vs control | 0.6599 | 0.4720 | 0.4642 | 0.4715 |
| HSCT | GMT (95% CI) | 8.36 (5.56-12.55) | 21.05 (14.63-30.3) | 14.32 (10.08-20.36) | 11.67 (8.28-16.44) |
| | P value vs control | 0.4622 | 0.1746 | 0.1909 | 0.8998 |
| Control | GMT (95% CI) | 9.33 (6.82-12.77) | 33.64 (21.29-53.14) | 22.19 (13.66-36.06) | 13.2 (8.62-20.21) |
| B Yamagata | | pre | Post1M | Post3M | Post6M |
| Chemotherapy | GMT (95% CI) | 10.88 (8.52-13.88) | 19.18 (13.27 -27.72) | 15.22 (10.7-21.66) | 13.14 (9.34-18.49) |
| | P value vs control | 0.9474 | 0.1306 | 0.9133 | 0.7767 |
| HSCT | GMT (95% CI) | 12.28 (8.34-18.07) | 21.05 (15.54-28.52) | 17.14 (12.99-22.63) | 14.70 (10.81-19.99) |
| | P value vs control | 0.3929 | 0.0008 | 0.0691 | 0.6407 |
| Control | GMT (95% CI) | 11.1 (8.05-15.29) | 64.98 (42.05-100.40) | 30.31 (20.93-43.91) | 14.14 (8.58-23.3) |
| Chemo, Chemotherapy; CI, confidence interval; GMT, geometric mean titer; CI, confidence interval; GMR, Geometric mean ratio; HSCT, Hematopoietic stem cell transplant; M, month; | | | | | |

Each test groups were compared with control group with two-tailed t-test. Statistical differences between control (n=20) vs Chemo (n=33) and control vs HSCT (n=27) calculated with two tailed t-test. Statistical significance was defined as *P < 0.05; **P < 0.01; ***P < 0.001; ****P < 0.0001.
